# HIV infection and domestic smoke exposure, but not human papillomavirus, are risk factors for esophageal squamous cell carcinoma in Zambia: a case–control study

**DOI:** 10.1002/cam4.434

**Published:** 2015-01-30

**Authors:** Violet Kayamba, Allen C Bateman, Akwi W Asombang, Aaron Shibemba, Kanekwa Zyambo, Themba Banda, Rose Soko, Paul Kelly

**Affiliations:** 1 Department of Internal Medicine, University of ZambiaLusaka, Zambia; 2 University of North Carolina at Chapel HillChapel Hill, North Carolina; 3 University of MissouriSt Louis, Missouri; 4 University Teaching HospitalLusaka, Zambia; 5 Barts and The London School of Medicine and DentistryLondon, United Kingdom

**Keywords:** Charcoal, firewood, HIV, HPV, esophageal cancer

## Abstract

There is emerging evidence that esophageal cancer occurs in younger adults in sub-Saharan Africa than in Europe or North America. The burden of human immunodeficiency virus (HIV) is also high in this region. We postulated that HIV and human papillomavirus (HPV) infections might contribute to esophageal squamous cell carcinoma (OSCC) risk. This was a case–control study based at the University Teaching Hospital in Lusaka, Zambia. Cases were patients with confirmed OSCC and controls had completely normal upper endoscopic evaluations. A total of 222 patients were included to analyze the influence of HIV infection; of these, 100 patients were used to analyze the influence of HPV infection, alcohol, smoking, and exposure to wood smoke. The presence of HIV infection was determined using antibody kits, and HPV infection was detected by polymerase chain reaction. HIV infection on its own conferred increased risk of developing OSCC (odds ratio [OR] 2.3; 95% confidence interval [CI] 1.0–5.1; *P *=* *0.03). The OR was stronger when only people under 60 years were included (OR 4.3; 95% CI 1.5–13.2; *P *=* *0.003). Cooking with charcoal or firewood, and cigarette smoking, both increased the odds of developing OSCC ([OR 3.5; 95% CI 1.4–9.3; *P* = 0.004] and [OR 9.1; 95% CI 3.0–30.4; *P* < 0.001], respectively). There was no significant difference in HPV detection or alcohol intake between cases and controls. We conclude that HIV infection and exposure to domestic and cigarette smoke are risk factors for OSCC, and HPV immunization unlikely to reduce OSCC incidence in Zambia.

## Introduction

Esophageal cancer is the eighth most common cancer globally and the sixth most common cause of cancer deaths 1. Southern Africa has the second highest incidence and mortality rates of esophageal cancer in the world 1, which makes it one of the major cancer burdens in the region. In Zambia, available registry data suggest that esophageal cancer is the fifth most common cancer, and is ranked fourth among the causes of cancer deaths 2.

Despite a well described increase in adenocarcinoma, esophageal squamous cell carcinoma (OSCC) remains the predominant type of esophageal cancer worldwide 3,4. There has been a steady increase in esophageal cancer cases in sub-Saharan Africa 5,6, although it is not clear if this is a real increase or a reflection of improved case detection. Esophageal cancer in sub-Saharan Africa develops at a considerably younger age than in other regions 7,8, and we reported that up to 27% of esophageal cancer patients seen in Zambia were below the age of 45 years 7. The risk factors for esophageal cancer in these young adults have not been established.

Sub-Saharan Africa is considered home to more than 60% of all human immunodeficiency virus (HIV)-infected people 9, and in Zambia the prevalence of HIV infection is 12.7% 10. The involvement of HIV infection in the development of SCC is uncertain. An analysis of registry data in the United States showed that the risk of developing esophageal cancer is higher among AIDS patients (standardized incidence ratio = 1.69; 95% confidence interval [CI], 1.37–2.07) 11, although the mechanism by which this occurs is not clear. There is also evidence that persons living with HIV infection are generally more susceptible to cancer than the general population 12. In 2009, Mlombe et al. published the findings of a small study in Malawi that showed increases in both esophageal cancer and Kaposi's Sarcoma (an HIV-associated cancer) 13. They offered no clear explanation for this observation, although this could have been due to HIV infection.

Three meta-analyses have concluded that human papillomavirus (HPV) infection has a role in the development of esophageal cancer 14–16, but there is also evidence against such a role 17,18. There is little evidence for the involvement of HPV in esophageal cancer in some areas in sub-Saharan Africa 19 and in a recent study in South Africa, HPV was shown to play a minor role in the pathogenesis of esophageal cancer 20.The prevalence of esophageal HPV infection in sub-Saharan Africa is unknown, although it was reported to be 24% among women with normal cervical cytology 21.

We undertook this case–control study to explore risk factors of OSCC in Zambia. We focused on HIV and HPV infections as infective agents that could be independently increasing the occurrence of OSCC, especially among young patients in Zambia. In addition, the lifestyle risk factors of smoking, alcohol, and exposure to wood smoke were evaluated.

## Materials and Methods

### Study design

This study was conducted at the University Teaching Hospital (UTH) in Lusaka, Zambia from October 2013 to May 2014. UTH is the only combined secondary and tertiary hospital in the country, and offers full time endoscopy services. The unit offers standard endoscopic evaluation using high-definition Pentax London, United Kingdom (EG2990i) gastroscopes. Patients attending this unit are referred from all parts of the country.

### Enrollment

All patients attending the unit during the study period were considered for enrollment. Consent for study participation was obtained before the endoscopy. Between October 2013 and May 2014, 67 patients with esophageal lesions suspected to be esophageal cancer gave consent to participate in the study. Of these, 61 had histologically confirmed esophageal cancer, including 58 (95.1%) OSCC and 3 (4.9%) adenocarcinoma. Adenocarcinoma patients were excluded from the analysis. Eight esophageal cancer cases were also excluded due to the unavailability of an appropriate control during the study period. Therefore, 50 OSCC patients were enrolled and matched by age (±3 years) and sex to 50 controls. Initial analysis showed that the association of OSCC with HIV infection was very close to being significant, which suggested that the sample size was too small to show an effect. To clarify the influence of HIV infection, we increased the power of the study by combining these data with 122 patients (27 cases and 95 controls) obtained from a preliminary case–control study conducted at the same unit between July 2011 and January 2012. This case–control study had the same inclusion and exclusion criteria as outlined above, but no frozen samples were available for HPV analysis. With these data sets merged, we conducted further statistical analysis of the HIV effect, as outlined in the results below. We, therefore, analyzed a total of 222 patients with 77 cases and 145 controls.

### Procedure

During the endoscopy, patients with lesions that were suspected to be esophageal cancer were designated as cases. A careful examination of the lesions was performed, and its location defined as follows: 15–24 cm, upper third; 24–32 cm, middle third; and 32–40 cm, lower third 22. We also took note of the degree of luminal occlusion. Six biopsies were obtained from these lesions and sent for histopathology. Status as a study case was only confirmed after invasive squamous carcinoma was reported by the team histopathologist (A. S.). Two additional biopsies were taken from the lesion, immediately placed in liquid nitrogen, and stored at −80°C for later determination of the presence of HPV infection. The next available sex- and age-matched patient (age within 3 years of the index case) was then enrolled as a control. Controls were completely normal upper endoscopic evaluations, and two esophageal biopsies were taken from the mid-esophagus of controls. As in the cases above, the biopsies were immediately placed in liquid nitrogen and taken to the laboratory to be stored at −80°C. We excluded patients with a history of any form of treatment for esophageal cancer or those unwilling to have an HIV test.

After the endoscopic procedure, 5–10 mL of venous blood was drawn from the study participants; serum was extracted and stored at −80°C for later determination of the presence of HIV antibodies.

A simple questionnaire was used to collect data on the lifestyle risk factors for esophageal cancer from both the cases and the controls. We also collected information about symptoms that were present.

### Histopathology

Six biopsies per participant were placed in formalin and sent to the histopathology laboratory. These biopsies were processed to 3 *μ*m sections, stained with hematoxylin and eosin for 1 h, and then examined using standard diagnostic criteria for OSCC. This included the presence of nuclear atypia and evidence of invasion.

### HIV testing

The frozen sera were thawed to room temperature and tested for the presence of HIV antibodies with Determine test kits (Alere Medical Co., Chiba, Japan) following the manufacturer's instructions. Unigold (Trinity Biotech plc, Bray, Ireland) kits were used as a confirmatory test for all the samples that tested positive with Determine. K. Z. conducted these tests and she was blinded to the allocation of the samples.

### HPV testing

DNA was extracted from frozen esophageal biopsy specimens using a Qiagen QIAmp DNA Mini Kit (Qiagen, Valencia, CA), according to the DNA Purification from Tissues protocol. A final elution volume of 200 *μ*L was used, and DNA extracts were frozen at −20°C until further testing. For each specimen, 10 *μ*L was used for the polymerase chain reaction (PCR) step of the DNA ELISA kit HPV SPF10 (LBP, Visseringlaan 25, ER Rijswijk, The Netherlands), which uses biotin-labeled SPF10 primers that target a conserved 65 bp region of the HPV L1 gene for broad-spectrum amplification. PCR products were stored at 4°C for less than 24 h before subsequent analysis. For each specimen, 10 *μ*L of PCR product was tested in the SPF10 ELISA and/or SPF10 LiPA25 (LBP) assay, performed per the manufacturer's instructions. The ELISA assay uses streptavidin-coated plates to capture biotin-labelled PCR products, followed by hybridization with a cocktail of labelled HPV-specific probes, which is detected with a conjugate and visualized by a substrate. The presence or absence of HPV DNA is determined by comparing the optical density of the PCR product to a cut-off value. The LiPA25 is a reverse line blot assay where denatured PCR products hybridize with specific oligonucleotide probes immobilized on membrane strips. Following hybridization and washing, streptavidin-conjugated alkaline phosphatase binds to biotinylated PCR products, and bands are visualized by a substrate. Due to limited ELISA reagents, nine specimens were solely tested using LiPA. The worker who performed the HPV testing (A. C. B.) was blinded to the case–control status of all specimens. In addition to kit controls, external quality assurance specimens were used as known positive controls. Positive controls and negative controls were used for each step (extraction, amplification, and detection), and the results of controls were as expected.

### Statistical analysis

Data were analyzed using STATA 13 (Stata Corp, College Station, TX). For categorical variables, the Fisher's exact test was used, and for continuous variables the Kruskal–Wallis rank tests were used. To analyze the effect of HIV infection in strictly matched case–control pairs, we also used McNemar's test. Odds ratios (ORs) with 95% CIs were derived to estimate the association between exposure variables and OSCC. A *P* value less than 0.05 was considered statistically significant. Using STATA, we performed a stepwise unconditional logistic regression to assess the relative contributions of different exposure variables (smoking, alcohol intake, HIV infection, HPV infection, exposure to household smoke, educational level, residence, marital status and occupation) to the risk of developing OSCC.

Ethical approval was obtained from the University of Zambia Biomedical and Ethics Committee (reference number 001-06-13).

## Results

### Baseline characteristics

The baseline characteristics of the cases and controls were similar (Table1). The mean age of the OSCC patients was 56.2 years, with the youngest patient being 28 years. The duration of symptoms was significantly shorter among the cancer cases than the controls (*P *=* *004), as shown in Table1.

**Table 1:** Characteristics of cases and controls in the initial case–control data set

	Cases (*n* = 50) *n* (%)	Controls (*n* = 50) *n* (%)	*P*
Sex M: F	36:14	36:14	1.000
Age mean (y)	56.2	56.1	0.856
Age below 45 years	14 (28)	16 (32)	0.828
Age below 60 years	28 (56)	29 (58)	1.000
Residence			
Urban	25/49 (51)	18/47 (38)	0.226
Rural	24/49 (49)	29/47 (62)	
Marital status			
Married	33 (66)	34/49 (69)	0.831
Not married	17 (34)	15/49 (31)	
Educational achievement			
Primary or lower	27 (54)	20/48 (42)	
Secondary or higher	23 (46)	28/48 (58)	0.234
Mean duration of symptoms (weeks)	17.7	194	0.004
Cooking with charcoal or firewood	34 (68)	18/48 (38)	0.004
Ever smoking	28 (56)	6/49 (12)	<0.000
Current smoking	19 (38)	0/49 (0)	<0.000
Ever alcohol	29 (48)	25/49 (51)	0.548
Current alcohol	19 (38)	11/49 (22)	0.123
Location of tumor			
Upper third	11 (24)		
Middle third	26 (28)		
Lower third	9 (18)		
Tumor classification			
Well differentiated	3 (7)		
Moderately differentiated	4 (9)		
Poorly differentiated	9 (20)		
Unclassified	30 (65)		
Esophageal occlusion			
No occlusion	4 (9)		
Partial occlusion	16 (37)		
Complete occlusion	26 (53)		

Denominators that are not exactly 50 have been indicated for clarity.

### OSCC characteristics

The full description of esophageal tumor characteristics was available in 46 of the 50 cases included in the analysis. These results showed that most of the tumors were either in the upper or the middle part of the esophagus and had some degree of luminal occlusion (Table1).

### Lifestyle risk factors for squamous cell carcinoma of the esophagus

Analysis of 50 OSCC cases and 50 controls showed that cooking with charcoal or firewood within the household increased the odds of developing OSCC (Tables1 and 2). Cigarette smoking (current and past) was also a strong risk factor for OSCC. There was no association between OSCC and residence (urban vs. rural), marital status, educational level or occupation (Table1).

**Table 2:** Lifestyle and biological risk factors of squamous cell cancer of the esophagus in the initial case–control data set

	Cases *n* (%)	Controls *n* (%)	Univariate		Multivariable^1^	
			OR 95% (CI)	*P*	OR 95% (CI)	*P*
HIV infection (serology)	11/49 (22)	8/49 (16)	1.5 (0.5–4.7)	0.610	2.8 (0.8–9.5)	0.092
HIV infection in patients less than 60 years	8/27 (30)	2/28 (7)	5.5 (0.9–56.9)	0.040	5.5 (1.0–27.7)	0.045
HPV infection (PCR)	2/44 (5)	1/48 (2)	2.2 (0.8–5.7)	0.605	2.2 (0.1–41.0)	0.596
Cooking with charcoal or firewood	34/50 (68)	18/48 (38)	3.5 (1.4–9.3)	0.004	3.0 (1.2–7.4)	0.021
Ever smoking	28/50 (56)	6/49 (12)	9.1 (3.0–30.4)	<0.000	8.0 (2.8–22.7)	<0.000
Current smoking	19/50 (38)	0/49 (0)	–	<0.000	–	–
Ever alcohol	29/50 (58)	25/49 (51)	1.3 (0.6–3.2)	0.548	0.3 (0.8–1.0)	0.057
Current alcohol	19/50 (38)	11/49 (22)	2.1 (0.8–5.7)	0.123	3.8 (0.8–18.1)	0.090

PCR, polymerase chain reaction; OSCC, esophageal squamous cell carcinoma; HIV, human immunodeficiency virus; HPV, human papillomavirus.

1 For the multivarible analysis, we included all the variables that could possibly be risk factors for OSCC. These included smoking, alcohol intake, HIV and HPV infection, exposure to household smoke, educational level, residence, marital status and occupation.

### HPV as a risk factor for OSCC

Of the 92 biopsy samples available for analysis, two cases and one control were positive for HPV (Table2). There was no significant difference in HPV infection between cases and controls in either univariate or multivariable analysis.

### HIV as a risk factor for OSCC, analyzed using the combined data set

Initial analysis suggested that there were more HIV-positive patients among cases than controls particularly when only patients under the age of 60 years were included (OR 5.5; 95% CI, 0.9–57; *P *=* *0.04) (Table2). To confirm if this important finding could be corroborated in a larger data set, we then analyzed combined data (from the current study and previous data) with an additional 122 records, for a total of 77 OSCC cases and 145 controls (Table3). There was no significant age difference between the two groups, with a median age of 55 years among the cases and 56 years among the controls (*P *=* *0.64). There were significantly more males among the cases (55; 71.4%) than the controls and (80; 55.1%), *P *=* *0.02. The presence of HIV infection did not differ between the two sexes, *P *=* *0.852. A total of 21 (27.3%) cases and 42 (54.5%) controls were below the age of 45 years (*P *=* *0.88). The presence of HIV infection did not differ significantly between cases and controls below the age of 45 years (OR 3.8; 95% CI, 0.8–20.5; *P* = 0.07). These data are not shown in the tables. We also found that 43 (55.8%) cases and 80 (55.1%) controls were below the age of 60 years (*P *=* *1.00) and there were significantly more HIV-positive patients among the cases than the controls (Table3). We then matched the 77 cases by age and sex (±3 years) to 77 of the controls (Table3). The association between OSCC and HIV infection was stronger in patients below the age of 60 years (OR 6.3; 95% CI, 1.5–36.4; *P *=* *0.006) (Table3).

**Table 3:** Human immunodeficiency virus as a risk factor for squamous cell carcinoma (combined data from the current study and from a previous case–control study)

Age	Cases *n* (%)	Controls *n* (%)	OR 95% (CI)	*P*
Entire data set				
All	18/77 (23)	17/145 (12)	2.3 (1.0–5.1)	0.03
Less than 60 years	14/43 (33)	8/80 (10)	4.3 (1.5–13.2)	0.003
Matched by age (±3 years) and sex^1^				
All	18/77 (23)	8/77 (10)	2.6 (0.1–7.5)	0.05
Less than 60 years	14/43 (33)	3/42 (7)	6.3 (1.5–36.4)	0.006

1 Analysis with McNemar's test, the results for all ages and those less than 60 years were similar: (OR 2.3; 95% CI, 1.01–5.01; *P *=* *0.04) and (OR 4.7; 95% CI, 1.45–15.1; *P *=* *0.005), respectively.

## Discussion

In recent years we have observed a worrying trend of increased occurrence of OSCC in young Zambian adults. Identifying the risk factors for these cancers is clearly a matter of great importance for gastroenterology in this region of southern Africa, and may unlock important clues to the etiopathogenesis of OSCC generally. In this paper, we report an investigation into the association of OSCC with various lifestyle risk factors, and the possible influence of HIV and HPV infection on the occurrence of OSCC among Zambian adults. We found that HIV infection independently increased the odds of developing OSCC. We further found that the use of firewood or charcoal as a means of cooking in the home, and cigarette smoking but not alcohol intake, increased the odds of developing OSCC. HPV infection does not appear to explain the occurrence of OSCC in Zambian adults.

The prevalence of HIV infection in the Zambian population is high (12.5% in very recent data) 10. There have been reports suggesting an increased risk of esophageal cancer among people living with HIV infection 11,23, although this has not been systematically investigated in areas heavily affected by HIV. Our initial analysis of 50 cases and 50 controls suggested an effect of borderline significance on multivariate analysis and in younger adults. Therefore, we decided to include data collected earlier in a similar case–control study conducted at the same unit. This increased the number of cancer cases to 77 and the results confirmed that HIV is associated with OSCC.

There is no clear consensus on the role of HPV in the development of esophageal cancer. Results from different studies have been inconsistent; Syrjänen et al. suggested that this could be because of geographic variations in the populations being studied 24. There is no baseline information on the prevalence of esophageal HPV infection in Zambia, but our results suggest that it is low and does not play a large role in the development of OSCC. Our findings are consistent with some studies from around the region, such as one from Kenya which showed that there was no HPV isolated in any of the esophageal cancer specimens evaluated 25. Therefore, unlike the situation in cervical cancer, HPV does not appear to explain the association between HIV and OSCC in younger adults. Disappointingly, these data suggest that widespread HPV immunization will not reverse the secular trend towards developing OSCC in young adults.

Exposure to wood smoke has previously been reported to increase the risk of developing esophageal cancer. In a study involving 99 cases and 223 controls in Brazil, exposure to woodstoves was found to increase the risk of esophageal cancer (OR 4.42; *P* < 0.001) 26. A recently published study from Kenya showed that poor socio-economic status and cooking with charcoal or firewood were risk factors for developing esophageal cancer 27. In the current study, we found that using firewood and charcoal for cooking in the home increases the odds of developing SCC. Zambia is an underdeveloped country with an estimated 80% of its population living in poverty. Only 19% of Zambian households have electricity, including 48% in urban and 3% in rural areas 28. Up to 41% of households in Zambia cook outdoors, again more in the rural (92%) than urban areas (37%). Firewood and charcoal are the most commonly used fuel for cooking in households without electricity, and rural areas rely mainly on firewood. These statistics show that a large proportion of Zambians are exposed to charcoal and firewood smoke throughout their lives, but the health risks of this exposure have not been ascertained. Exposure to inhaled wood smoke could also explain the reported higher incidence of esophageal cancer among people of low socio-economic status. This requires further study, because it is not clear from our data how this extra risk is mediated, whether by inhalation, ingestion on cooked foodstuffs, or direct contact with cooking utensils. We are inclined to believe that this and other risks from wood smoke exposure during cooking (respiratory disorders) could be avoided by widespread introduction of alternative energy sources, but this is beyond the scope of the present study.

Established risk factors for esophageal cancer include smoking, alcohol 29–32 and advancing age 3. Risk factors for adenocarcinoma include Barrett's esophagus, obesity and the use of anti-acids 4. There are no published data on the genetics and risk factors for OSCC in Zambia, although in studies within the region (South Africa and Uganda), smoking has been reported to play a major role in esophageal cancer 3,31,33. However, there is a need to further evaluate the relationship between gene susceptibility and lifestyle risk factors such as smoking 34. We found a highly significant association between cigarette smoking and OSCC. We collected information on patients who were still smoking at the time of enrollment and those who had stopped smoking. None of the controls were current smokers; therefore, we did not calculate the ORs or include this variable in the multivariate analysis. However, 38% of cases smoked at the time of enrollment, a much higher proportion of smoking than controls.

Reilly 35 reported a possible correlation between esophageal cancer and the consumption of home-produced alcoholic drinks in Zambia. However, we have not found evidence in support of this, because neither current nor past use of alcohol was associated with OSCC in our study.

This is the first study that prospectively collected data on the age distribution of esophageal cancer patients in Zambia, and we have found that 28% were below the age of 45 years. This is similar to our previous findings in gastric cancer cases in which 21% were below 45 years 36, and an earlier retrospective report on esophageal cancer in which it was 27% 7. Taken together, these data from one central, busy endoscopy unit indicate that there is an emerging problem with gastrointestinal cancers in young Zambian adults.

The outcome of esophageal cancer patients in limited resource countries such as Zambia tends to be very poor. One of the postulated reasons is due to late presentation for medical assistance. For the 50 OSCC patients enrolled into this study, their symptoms had been present for an average of 17.7 weeks, ranging from 1 to 52 weeks. This clearly shows that there is a need to sensitize this population to seek medical assistance as soon as symptoms of dysphagia start to develop, to improve the treatment outcomes.

In conclusion, HIV infection and exposure to domestic and cigarette smoke are risk factors for OSCC. HPV infection and the use of alcohol do not appear to play a large role in OSCC in Zambia.
